# Subtle Inefficiencies in Everyday Tasks are Significantly More Frequent in People with Mild Cognitive Impairment and Associated with Episodic Memory Performance: Implications for Clinical Practice and Research

**DOI:** 10.1002/alz.091061

**Published:** 2025-01-03

**Authors:** Moira Mckniff, Marina Kaplan, Sophia L. Holmqvist, Molly B. Tassoni, Stephanie M. Simone, Tania Giovannetti

**Affiliations:** ^1^ Temple University, Philadelphia, PA USA

## Abstract

**Background:**

Mild functional difficulties begin in mild cognitive impairment (MCI) and precede functional disability, but people with MCI generally perform at ceiling on performance‐based tests of everyday function. This study examined whether inefficient reaching, touching, and extra movements (i.e., micro‐errors) on a performance‐based test (Naturalistic Action Test [NAT]) are more frequent in people with MCI vs healthy cognition and whether micro‐errors are associated with cognitive abilities, reflecting cognitive failures.

**Method:**

Community dwelling older adults over the age of 65 (N = 89; MAge = 73.54; SD = 6.9) completed tests of global cognitive status, episodic memory, executive functioning, language, and attention and were classified as having healthy cognition (n = 74) versus MCI (n = 15) using Jak/Bondi criteria. Participants also completed the NAT, which includes a breakfast and a lunch task, at two time‐points separated by 4‐6 weeks. NATs were scored for subtle, inefficient actions (i.e., micro‐errors) by coders who were blind to cognitive status and timepoint.

**Result:**

An ANCOVA (controlling for age) showed participants with MCI made significantly more micro‐errors than participants with healthy cognition at both timepoints [Timepoint 1: F (1, 87) = 7.368, p = .008, η2 = .079; Timepoint 2: F (1, 87) = 15.167, p < .001, η2 = .151]. Micro‐errors at time 1 were correlated with global cognitive status [r = ‐.229, p = .031], episodic memory [r = ‐.223, p = .036], and micro‐errors at time 2, but were not correlated with measures of executive function, language, or attention. A Spearman’s rank order correlation to establish test‐retest reliability did not reach our .7 cut‐off, though the correlation was significant (r (87) = .465, p < .001), and both time‐points did not significantly differ from each other (p = .086)

**Conclusion:**

Although people with MCI are functionally independent, they make more inefficient actions (micro‐errors) than healthy controls on highly familiar everyday tasks, and this difference was stable over time. Micro‐errors, which correlations suggest reflects cognitive failures, may be useful markers for early detection of MCI, identification of risk for conversion to dementia or functional disability, and may be used as functional outcomes in clinical trials.

